# An Investigation Into the Unconscious Influence of Mortality Salience Upon Sentencing Decisions

**DOI:** 10.1177/00332941241295971

**Published:** 2024-10-23

**Authors:** Bethan Robinson, Daniel R. Stubbings, Joseph L. Davies, Deiniol Skillicorn

**Affiliations:** Department of Applied Psychology, School of Sport and Health Sciences, 11352 Cardiff Metropolitan University, Cardiff, UK

**Keywords:** Terror management theory, mortality salience, legal decision making, subliminal priming

## Abstract

This study aimed to explore if unconscious awareness of death influences the harshness of offender sentencing. According to Terror Management Theory death is anxiety-provoking, and self-esteem and a belief in a shared cultural worldview keep anxiety at bay. When these factors are challenged then death awareness increases. These dynamics could be relevant in a court setting in which judges have to make decisions regarding offenders who may have different world views and in cases that trigger the awareness of mortality. We used subliminal priming to activate the awareness of death and recorded the effect it had on decision-making against a hypothetical offender. Participants (*N* = 303) were recruited and randomly assigned to either an experimental mortality condition or a neutral control condition. Analysis revealed that death-related subliminal priming brought about harsher sentencing effects than the control. The results suggest that subconscious awareness of death may bias decision-making when sentencing.

## Introduction

Integral to the concept of a fair criminal justice system is the precept that guilt, or innocence, is determined by an objective and effective legal process (Collins, 1997). Fair trials are a fundamental part of a just society and help prevent the miscarriage of justice. However, the underpinnings of a fair trial rely on judges and jury remaining impartial and independent of external pressures when carrying out judicial duty. Despite the concept of a fair and impartial system, many legal scholars and scientific researchers concur that this assumption is somewhat flawed. Kassin and Wrightsman (1988) argue that judges are susceptible to biases which can manifest in their final verdicts. Recent literature has demonstrated that race, age, and sex (Nunez et al., 2007), emotions and attitudes (Devine, 2012), and awareness of one’s mortality (Hayes et al., 2010), can all impact legal outcomes.

Terror Management Theory (TMT; Greenberg et al., 1986) is focused on understanding the impact that awareness of mortality has on human motivation, emotion, and behaviour. Immortality is unattainable, so as a species, our innate desire for self-preservation and survival can be problematic (Becker, 1973). According to TMT, a conscious and/or unconscious awareness of one’s inevitable death can activate intense anxiety (Gordillo et al., 2017). We combat this anxiety by having an allegiance to a cultural worldview that includes standards for socially valued behaviours and beliefs (Arndt et al., 2005). Worldviews are subjective and can be influenced by and categorised under many competing factors including but not limited to; political affiliation, religious beliefs, cultural association, gender ideology, and/or social class (Kyle, 2018). When mortality is made salient consciously or unconsciously, individuals tend to psychologically gravitate towards the dogma of their worldview to protect their psyche from the anxiety triggered by mortality awareness (Rosenblatt et al., 1989). This psychological proclivity provokes individuals to respond more favourably to those who share the same worldview but adversely to those who hold conflicting beliefs (Arndt et al., 2005; Bandt-Law & Krauss, 2017; Greenberg et al., 1990; Heen et al., 2016).

Over four hundred studies have provided empirical support for TMT hypotheses (Greenberg et al., 2008). In laboratory settings, the hypothesis has been tested by presenting death-related images, asking participants to think about their death, or interviewing them in proximity to locations that may elicit death-related thoughts, such as graveyards. The strongest effects have been seen when an individual contemplates their death as opposed to the death of a loved one or mortality in general (Greenberg et al., 1994). For example, American participants who were asked to contemplate their death provided positive evaluations to those with the same nationality but were increasingly hostile towards international citizens who threatened and did not appear aligned with their cultural worldview (Motyl et al., 2009).

### TMT and Legal Decision Making

During criminal proceedings, legal decision-makers are faced with offenders who have violated cultural guidelines and disobeyed the law (Bandt-Law & Krauss, 2017). In light of TMT theory, one would expect that when mortality is made salient and/or when an offender presents as having a different worldview to the legal professional legal decision makers would be more punitive towards offenders (Schimel et al., 1999). Rosenblatt et al. (1989) were the first to explore this phenomenon and conducted a series of experiments using hypothetical cases, with one of these experiments including a sample of 22 judges. In their experiment, the authors assigned judges to a mortality-salient condition, where they would be reminded of their mortality and a control condition. For both conditions, judges were required to set a bond for an alleged prostitute and provided a hypothetical case based on this defendant. They found that participants in the control condition were more likely to set a higher bond than participants in the control condition.

In another study, Pickel and Brown (2002) simulated a criminal trial and examined the influence of mortality salience on juror decision-making. Mock jurors were asked to recommend a prison sentence for an offender caught driving under the influence of alcohol. Mortality salience was incorporated into the prosecutor’s closing argument by asking the ‘jurors’ to consider how it would feel to be the victim of a fatal car crash. Participants exposed to the mortality manipulation were more likely to recommend a longer prison sentence, compared to control participants who had not been exposed to death. It is noted, however, that Cook et al. (2004) obtained a similar pattern of results in the context of an armed robbery and attempted murder trial. Exposure to death reminders provoked more punitive responses and more severe verdicts to those who had violated the law.

The notion that an awareness of death provokes harsher reactions has received cross-cultural, empirical support (Burke et al., 2010; Dechesne et al., 2003; Florian et al., 2001). The influence of mortality salience on sentencing outcomes has been replicated in several legal scenarios, with a variety of victims, defendants, and crime types. Florian et al. (2001) explored the influence of mortality salience on guilty verdicts across several criminal offenses, including traffic violations, burglary, robbery, forgery, and fraud. Across all crime types, individuals were significantly more likely to convict alleged offenders when reminded of death. Furthermore, a breadth of TMT research has focused on inducing mortality by explicitly asking participants to contemplate their own death (DeWall & Baumeister, 2007; Maxfield et al., 2007). Despite a lack of literature investigating the unconscious manipulation of mortality, research has indicated that subtle, subliminal provocations of death can trigger mortality salience reactions (Burke et al., 2010). Arndt et al. (1997) successfully induced mortality outside of conscious awareness by subliminally presenting death-related stimuli. The results revealed that the subliminal presentation of the word *death* increased the accessibility of death-related thoughts and provoked increased defence for participants’ cultural worldview.

While few attempts have been made to induce awareness of mortality outside of conscious awareness, little is known about the relationship with legal decision making and recent attempts to replicate mortality salience effects have failed (Treger et al., 2023). Furthermore, many studies conducted in this area have relied on small sample sizes (Arndt et al., 2005; Lieberman et al., 2014), decreasing the scope of generalisation, or are affected by confounding variables (Pickel & Brown, 2002). The current study aimed to explore mortality salience in a specific case as an initial step to be further developed considering cross-cultural and demographic measures and biases. It also sought to build upon the limitations of existing TMT research while addressing a gap in the field by exploring the subliminal activation of unconscious processing of mortality and its impact on criminal sentencing decisions. A subliminal priming task to manipulate mortality salience in the context of a crime vignette was presented to participants. Participants were required to state the length of the sentence following the presentation of the crime vignette. This method of exploring subliminal priming has been modeled on prior research that has shown this method of exposure to be effective in causing changes in participant response (Arndt et al., 1997; Kawakami et al., 2018; Landau et al., 2004; Mahoney et al., 2014). It is expected that when people are subliminally primed to unconsciously think about death they are likely to sentence offenders for longer.

## Methodology

### Design

A between-subjects design was applied with one independent variable; participants assigned condition, and number of death-related words as the dependent variable. The assigned condition was a categorical variable with two levels: a ‘mortality salience prime’ condition (the word “Death”), and a ‘no mortality salience prime’ condition (the word field). There were two dependent variables. These included the sentence length prescribed to the offender in the forensic scenario which was measured continuously. The second dependent variable was the number of death-related words which was a continuous variable.

### Participants

An opportunistic sampling method using a non-probability approach was used to collect participants in the United Kingdom. Participants were recruited through an anonymous online link to the study which was distributed through a social media advert on multiple platforms (Facebook, Twitter, Instagram, WhatsApp, Snapchat). Participants were required to be aged eighteen years or older to participate. Participants were randomly assigned to either the ‘mortality salience prime’, or ‘no mortality salience prime’ condition. A total of 339 participants were recruited. Participants who provided a partial response (29 participants) or withdrew from the study (7 participants) were excluded. The final sample comprised of 303 participants with 161 participants in the experimental prime and 142 in the control.

### Materials

#### Subliminal Priming Task

**Figure 1.**
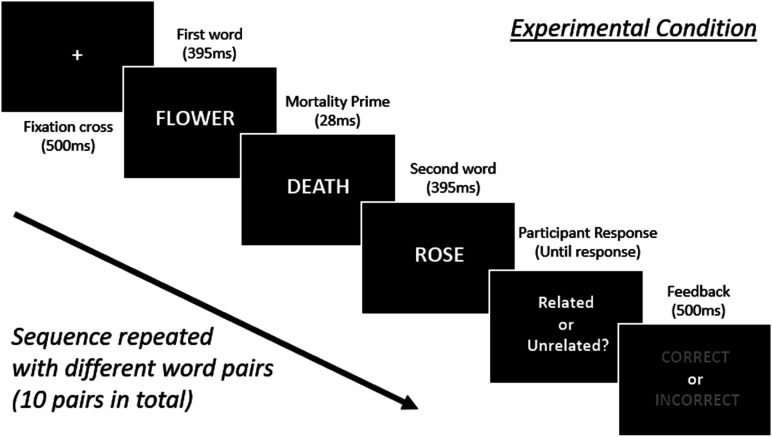
An example sequence for the mortality prime (experimental) condition.

A subliminal priming task was created on OpenSesame replicating the methodology of Mahoney et al. (2014). OpenSesame is a program used to create online experiments for psychology and neuroscience researchers (Mathôt et al., 2012). Utilising a JATOS server (Lange et al., 2015), participants had direct access to the study via an anonymised link. Participants only took part in the conditions they were allocated to. During the task, two words appeared sequentially on the monitor and participants indicated whether the words were related (by pressing the “Z” key) or not related (by pressing the “M” key). For example, the word pair ‘Flower and Rose’ (related – “Z” key) or ‘Flower and Sneaker’ (not related – “M” key). Participants were presented with 10 consecutive word pairs (5 related and 5 unrelated pairs), which were shown in a random order. In between these word pairs a subliminal word was presented. In the mortality prime condition (experimental), the word ‘death’ was presented and in the control condition the word ‘field’ was used. In line with the wider cognitive literature (Willenbockel et al., 2013; Quiroga et al., 2008), the words ‘death’ and ‘field’ were presented at a length of time below the ability for conscious recognition (28 ms). After each word pair, participants were told whether their response was correct or incorrect. An example sequence can be seen in Figure 1.

#### Word Fragment Task

A manipulation check was used to assess whether the subliminal priming task had successfully induced MS and subsequent death-related thoughts (Schimel et al., 2007). The accessibility of death-related thoughts was measured through a Word Fragment Task (Schimel et al., 2007). The task comprised 20-word fragments, whereby two letters from each word were replaced with blank spaces (Appendix B). Six-word fragments could be filled out with either non-death-related words or death-related words (buried, dead, grave, killed, skull, coffin). For example, ‘S K _ _ L’ could be completed as the death-related word ‘skull’ or the neutral word ‘skill’. A death-thought accessibility score was calculated by the number of death-related word completions (0–6 words).

#### Crime Vignette

The crime vignette template used was the London Bridge terror attack from 2019 (see Appendix A) but the name was changed from Usman Khan to Ahmed Hussain, the date to 2020 and the location to Manchester. Participants were required to record a sentencing decision, between 15 to 25 years based on the crime vignette. This sentencing scale was established based on sentencing guidelines in the UK courts (CPS, 2019) for committing a double homicide.

### Procedure

The project was approved by the Cardiff Metropolitan University Ethics Committee (Approval Reference: PGT-2772). To recruit participants an advert was distributed across various social media platforms (Facebook, Instagram, Twitter, and WhatsApp). The social media advert briefly explained the study, the eligibility criteria, and how to access the study. If participants were interested in completing the study they clicked on the link provided. Upon accessing the study participants were presented with an information sheet and consent was documented through a tick box. Participants had to indicate whether 10 different word pairs were related or not related. After each word pair participants were told whether their response was correct or incorrect.

Following the completion of the subliminal priming task, participants were asked to complete the Word Fragment task. Participants were instructed to complete the task as quickly as possible and fill in the blank spaces with the first word that came to mind. Then, all participants regardless of the allocated condition were asked to read the crime vignette. This provided information about a terrorist attack and the perpetrator’s criminal history. The vignette was identical in both conditions. Based on the vignette, participants then had to provide a sentencing decision for the offender (15–25 years). A debrief sheet was created with a detailed explanation of the study, the expected outcomes, and contact details if in need of support. At this point, participants were informed of the condition they had been subject to.

### Method of Analysis

A number of statistical methods of analysis were conducted in the current study, using the Statistical Package for Social Sciences (SPSS, Version 28). To discover whether the Subliminal Priming Task was able to induce the accessibility of death-related thoughts, an independent samples *t* test was conducted. Another independent samples *t* test was conducted to compare sentence length in each condition of the Subliminal Priming Task. To assess the potential mediation of exposure to death-related words, a one-way ANCOVA was run to determine the effect of subliminal priming conditions on sentence length with exposure to death-related words as a covariate. A multiple linear regression was performed to predict sentence length from the Subliminal Priming task and the Word Fragment Task. To explore potential moderation, a hierarchical multiple regression was conducted to determine whether the relationship between the number of death-related words on the Word Fragment Task and sentence length was moderated by the Subliminal Priming Task.

## Results

An independent samples *t* test was conducted to discover whether the subliminal priming task was able to induce the accessibility of death-related thoughts. There was a significant difference in the number of death-related words in the experimental condition compared to the control; *t* (301) = 9.67, *p* < .001. Participants in the experimental condition provided more death-related words (*M* = 3.98, *SD* = 1.81), on average than participants in the control condition (*M* = 2.0, *SD* = 1.73). This indicates that mortality was made more salient in the experimental condition.

Another independent samples *t* test was conducted to compare sentence length between the experimental condition and control conditions. There was a significant difference in sentence length for those in the experimental condition compared to the control condition; *t* (301) = 8.88, *p* < .001. Participants who were exposed to death stimuli provided longer sentences (*M* = 26.27, *SD* = 4.78), on average, compared to those in the control condition (*M* = 21.02, SD = 5.50).

A one-way ANCOVA was run to determine the effect of subliminal priming conditions on sentence length controlling for exposure to death-related words. After adjustment for the number of death words exposed to participants, there was still a statistically significant difference in sentencing lengths between the experimental condition and the control conditions, *F* (1, 300) = 21.283, *p* < .001, partial η^2^ = .066. Post hoc analysis with a Bonferroni correction suggested that sentence lengths were significantly greater in the MS Prime condition versus the No MS Prime condition (*M*_diff_ = 2.831, 95% CI [−1.623, 4.038], *p* < .001).

A multiple linear regression was performed to predict sentence length from the priming task (exposure to death) and the word fragment task (number of death-related words). The model emerged statistically significant, *F* (2, 300) = 80.59; *p* < .001. The model accounted for 34.9% of the variance (Adjusted *R*^2^ = .35). Both variables were statistically significant to the prediction of sentence length, *p* < .001. A semi-partial correlation was conducted to determine which variable contributed more strongly towards sentence length, the subliminal prime task (exposure to death) or the word fragment task. 18.8% of the sentence length variance was uniquely explained by the IVs and 16.1% was explained by the combination of both IVs. The subliminal prime task (exposure to death) was the strongest predictor of sentence length, accounting for 14.2% of the variance (sr^2^ = .142), whilst the word fragment task uniquely explained 4.6% of the variance (sr^2^ = .046). Unconscious exposure to MS was the most influential variable for sentence length.

A moderation analysis was then used to determine whether the relationship between the number of death related words (word fragment task) and sentence length was moderated by the subliminal priming task. A hierarchical multiple regression revealed a significant moderator effect of experimental manipulation (allocated condition), which explained an additional 34.9% of the total variance, *p* < .001. Exposure to death significantly mediated the relationship between the number of death related words and the sentence length. The outcome of the word fragment task and mock court case (sentence length) depended on whether participants were exposed to death or not (control or experimental condition).

## Discussion

The aim of the current study was to explore the subliminal influence of mortality salience upon sentencing decisions. Findings from the current study were that subliminal priming of mortality salience influenced accessibility of death-related thoughts, and the length of sentencing ascribed to a crime vignette. The influence of subliminal priming of mortality salience on sentencing length was still present even when controlling for the number of death-related words expressed to participants on the Word Fragment Task.

Based on previous TMT research (Greenberg et al., 1986; Mahoney et al., 2014), it was hypothesised that individuals who were subliminally primed with death-related stimuli would be more punitive in their proposed sentence lengths. This hypothesis was validated by the study findings with significant differences in sentence length found between the mortality salience prime and control condition. Participants in the mortality salience condition suggested harsher sentence lengths than participants in the control condition. Taking previous TMT research into account we postulate that this effect is the result of an increased awareness of death, which caused heightened anxiety amongst participants exposed to more death-related stimuli (Gordillo et al., 2017), although caution must be taken when interpreting our results in this way, given we did not measure self-reported affect.

Whilst the subliminal priming task significantly impacted sentence length, the nature of the crime used in the court case may have induced further death-related thoughts and helped sustain mortality salience effects. The crime vignette included the loss of life, and so thoughts of death may have been aroused simply by the description of the terrorist attack. As noted by Lerner et al. (2003), terrorism is the optimum crime for investigating mortality salience effects as it poses a contemporary threat that evokes fear. Terrorist attacks threaten mortality so legal decision makers may take a subjective stance based on the perceived likelihood of being a victim (Pyszczynski et al., 2008). For instance, in the years following the World Trade Centre attacks, reminders of death increased support for anti-terrorist policies that would kill thousands of innocent people in the Middle East (Landau et al., 2004). Furthermore, despite a significant difference in sentence length between conditions, the average sentence length in the control group was still relatively long. A plausible explanation for this could be that the crime vignette alone induced a fear of death.

### Implications

Our finding that mortality salience exposure negatively influences sentencing decisions is also consistent with previous research showing the negative impact of mortality salience on legal decision-making (Cook et al., 2004; Dechesne et al., 2003; Florian et al., 2001; Rosenblatt et al., 1989). Our findings highlight the potential bias that judges may be susceptible to when determining sentencing for offenders. Legal decision-makers may be unaware of the biases elicited by unconscious concerns about their mortality (Kirchmeier, 2008). Thus, it is important to recognise the potential impact that our innate biological predisposition for self-preservation has on the fair judiciary process.

Alongside fair process, the results pose significant implications for trial strategy and deliberation. Attorneys may want to focus on TMT to enhance the effects of mortality salience during trial proceedings. Prosecutor attorneys may want to focus on heightening judges’ awareness of mortality through death related case presentations, opening statements and closing arguments (Howe, 2009). Lieberman (2009) suggests highlighting case facts that would threaten a judges’ worldview or posing questions to lead judges to contemplate their own mortality. Judges must also be reminded of the importance of remaining rational and logical, whilst following legal rulings, when making sentencing decisions (Simon et al., 1997). Future research is needed to uncover if it is possible to mitigate bias in judges and whether an insight into the influence of unconscious bias can reduce the effect it has on sentencing decisions.

### Limitations and Future Research

The participants in this study were members of the general public who knew that their decisions would not have consequences for the defendant. It is very possible that experienced judges make their decisions in a more objective manner. The crime vignette was short and lacked the detail of a real-life homicide trial and it could be argued that in a real case conscious and unconscious death anxiety would be greatly heightened, thus amplifying the effects observed above. However, establishing ecological validity in laboratory research is a well-known challenge in the social sciences and the cognitive, social, and emotional processes maybe different in real-world settings (Chae, 2010).

Another important limitation to consider is the use of static conditions in the subliminal priming task. In this study participants were either subconsciously exposed to mortality stimuli or neutral stimuli. However, Lieberman (2009) notes that morality salience is not an “either or” phenomenon, rather it is something that occurs in varying degrees. Depending on the nature of criminal conduct, judges’ may be exposed to subtle or extremely salient reminders of death. For instance, a life-threatening criminal act, such as murder, will include more prominent death related references as opposed to a drug possession offence (Dewa et al., 2014; Leippe et al., 2017). Thus, utilising terrorism in the vignette may have enhanced death-related thoughts amongst participants and intensified overall effects. Given the considerations for crime type, future research would need to explore how the type of crime impacts sentencing decisions and whether the same effects can be seen in a trial that involves no death or violence.

The study did not collect participant demographic information of the participants therefore it was impossible to comment on the overlap in cultural alignment between the participants and the hypothetical case example. It is likely that participants who have a more favourable worldview overlap with the defendant may not give as harsh of a sentence (Nunez et al., 2007). Previous research has shown that decision makers are more punitive towards criminals who do not share the same demographic characteristics (Sommers & Ellsworth, 2000) but it would be ideal to compare all factors within the one study and analysis.

## Conclusion

This study continues to support the notion that unconscious awareness of mortality salience has the potential to increase the harshness of offender sentencing. This effect is explained in the context of TMT. In cases where the offender appears to have a different cultural worldview from the norm, and the person doing the judging has a heightened level of mortality salience, sentences tend to be longer. The implication is that judges who are more anxious about death unconsciously, have been triggered to think about death by the case, and have a different cultural worldview than the offender, could be biased into giving longer sentences. Future research needs to determine if this effect holds for practicing judges.

## Data Availability

Data is not available for sharing.

## Appendix A

### Court Case Example

Please read the following Court Case and then decide on the length of sentencing (15–25 years) Ahmed Hussain is a 28-year old British National of Pakistani descent. Hussain has previously been involved with the Islamist extremist group, Al-Qaeda. In 2012, he was found guilty of terrorist-related offences including involvement in a plot to bomb the London Stock Exchange, the Houses of Parliament and the US embassy. He was ordered to serve a minimum sentence of 8 years in prison. In December 2018, he was released from prison early with an electronic tag. In January 2020, Ahmed Hussain attended an offender rehabilitation scheme in Central Manchester. During this, the convicted terrorist went on a deadly rampage. Hussain threatened to blow up the building by detonating what turned out to be a fake suicide vest. Armed with two kitchen knives, Hussain then began stabbing people inside the building. Several members of the public were injured as they attempted to restrain him. Two members of the public were killed, and several others were seriously injured. Based on the case you have just read, please provide the length of sentence you would give to the perpetrator, Ahmed Hussain? (15–25 years)

## Appendix B

### Word Fragment Task

**Table:**

Please complete the following task by filling letters in the blanks to create words. Please fill in the blanks with the first word that comes to mind. Write one letter per blank. Some words may be plural.	
1. *BUR _ _ D*	11. *CHA _ _*
2. *PLA _ _*	12. *KI _ _ ED*
3. *WAT _ _*	13. *TAB _ _*
4. *DE _ _*	14. *W _ _ DOW*
5. *MU _ _*	15. *SK _ _ L*
6. *_ _ NG*	16. *TR _ _*
7. *B _ T _ LE*	17. *P _ P _ R*
8. *M_ J _ R*	18. *COFF _ _*
9. *FL _ W _ R*	19. *POST _ _*
10. *GRA _ _*	20. *R _ DI _*
